# Health-related quality of life in Marfan syndrome: a 10-year follow-up

**DOI:** 10.1186/s12955-020-01633-4

**Published:** 2020-12-01

**Authors:** Thy Thy Vanem, Svend Rand-Hendriksen, Cathrine Brunborg, Odd Ragnar Geiran, Cecilie Røe

**Affiliations:** 1 Institute of Clinical Medicine, Faculty of Medicine, University of Oslo, Oslo, Norway; 2 Department of Cardiothoracic Surgery, Oslo University Hospital (OUH), Oslo, Norway; 3 TRS National Resource Centre for Rare Disorders, Sunnaas Rehabilitation Hospital, Nesoddtangen, Norway; 4 Oslo Centre for Biostatistics & Epidemiology, Oslo, Norway; 5 Department of Physical Medicine and Rehabilitation, OUH, Oslo, Norway

**Keywords:** Marfan syndrome, Follow-up, Health-related quality of life, SF-36

## Abstract

**Background:**

Marfan syndrome, a rare hereditary connective tissue disorder caused by mutations in fibrillin-1, can affect many organ systems, especially the cardiovascular system. Previous research has paid less attention to health-related quality of life and prospective studies on this topic are needed. The aim of this study was to assess changes in health-related quality of life after 10 years in a Norwegian Marfan syndrome cohort.

**Methods:**

Forty-seven Marfan syndrome patients ≥ 18 years were investigated for all organ manifestations in the 1996 Ghent nosology and completed the self-reported questionnaire, Short-Form-36 Health Survey, at baseline in 2003–2004 and at follow-up in 2014–2015. Paired sample *t* tests were performed to compare means and multiple regression analyses were performed with age, sex, new cardiovascular and new non-cardiovascular pathology as predictors.

**Results:**

At 10-year follow-up: a significant decline was found in the physical domain. The mental domain was unchanged. Older age predicted a larger decline in physical health-related quality of life. None of the chosen Marfan-related variables predicted changes in any of the subscales of the Short-Form 36 Health Survey or in the physical or the mental domain.

**Conclusion:**

Knowledge of decline in the physical domain, not related to organ affections, may be important in the follow-up of Marfan syndrome patients.

## Introduction

Marfan syndrome (MFS) is a rare hereditary connective tissue disorder (HCTD), caused by mutations in fibrillin-1 (*FBN1*) (OMIM 134797). The diagnosis is based on clinical criteria and DNA sequencing. Population based prevalence has been reported between 4.6 and 10.2 per 100,000 [1–3]. MFS can affect many organ systems, among them the cardiovascular system with manifestations such as aortic dilatation and aortic dissection; the ocular system; the skeletal system; the dura mater; the pulmonary system and the skin and integuments. Organ manifestations seem to progress throughout life [4–6]. Although life expectancy has increased since the 1970’s [7], it is still shortened in MFS patients, mainly due to aortic and other cardiovascular affections [1, 8]. Research on MFS has focused on organ affections, molecular pathogenesis and surgical and medical management [9], with less attention to health-related quality of life (HRQoL) [10]. As treatment has improved and life expectancy has increased, more knowledge is needed regarding HRQoL and psychosocial consequences of living with this chronic condition.

Studies on HRQoL in MFS patients are mainly designed as cross-sectional studies, the quality are varying, the results diverging and in almost half of the studies, the participants do not have a verified diagnosis of MFS [10, 11]. Most studies report reduced HRQoL in MFS patients compared to the general population or other controls, but the study populations have often been small, the response rates low and different methods for assessing HRQoL have been used [12–20].

The Short Form-36 Health Survey (SF-36) from the Medical Outcomes Study [21] has been the most frequently used tool for assessing HRQoL in adults with MFS [10]. SF-36 measures self-reported health status and comprises eight subscales: physical functioning, role-physical, bodily pain, general health, vitality, social functioning, role-emotional and mental health. The mental component summary (MCS) and the physical component summary (PCS) scores are calculated from these eight subscales. Other instruments that have been used for assessments of HRQoL in the adult MFS population are the SF-12 (a shorter version of SF-36) [22], The Ferrans and Powers QoL index [23], and the Nottingham Health Profile [24], reflecting variations of the dimensions in HRQoL.

Reduced HRQoL in MFS patients has been associated with mental fatigue [25] and difficulties in attentional and memory abilities [20]. One study found good correlations between some sleep complaints and reduced HRQoL in some of the subscales of the SF-36 [12]. Most studies have not found associations between the cardiovascular severity of the syndrome and low HRQoL [16, 17, 26]. Only one study found that disease-related factors, including cardiovascular manifestations, affected HRQoL in MFS patients [27]. Severe scoliosis has been weakly related to a reduced physical HRQoL [17]. Better HRQoL has been associated with insurance and employment status [26]. Pain has been reported prevalent in 47–91% of MFS patients [12, 28]. One study found associations between physical and mental health functioning and pain-related disability [29]*.*

In our baseline study from 2003–2004 [16], reduced scores were found in all the subscales of the SF-36 and for MCS and PCS in the Norwegian MFS study cohort, compared to the general Norwegian population. Increasing age in MFS patients was associated with reduced HRQoL in two subscales, bodily pain and physical functioning. No associations were found between any of the subscales and sex, body mass index, ascending aortic surgery or joint hypermobility.

To our knowledge, only one observational pilot study has presented HRQoL follow-up data in a small MFS study population, 1 year after a 3-week rehabilitation program, reporting improved HRQoL on one subscale of the SF-36, role-physical, and on one subarea of the Nottingham Health Profile, emotional reaction [30].

With the contradictory associations between organ pathology and HRQoL, prospective studies are warranted.

The aim of this 10-year follow-up study was to assess changes in the eight subscales of the SF-36 and changes in MCS and PCS. Secondly, we wanted to explore whether age, sex, development of new cardiovascular pathology or other new severe organ pathology predict decline in any of the subscales or in MCS and PCS.

## Materials and methods

This study is based on a Norwegian MFS cohort [31], 18 years of age or older. Patients with presumed MFS were recruited through letters to all adults registered as having MFS at TRS National Resource Centre for Rare Disorders; through information letters delivered to MFS patients at the Department of Cardiothoracic Surgery at Oslo University Hospital or through information published in the magazine for the Norwegian Marfan Association. All participants were investigated in 2003–2004 (baseline) for all the organ systems described in the 1996 Ghent nosology (Ghent-1) [32], and the SF-36 questionnaires were completed [16]. All received a report with recommendations for future follow-up of Marfan-related manifestations after the baseline study. *FBN1* was sequenced in all participants and whole exome-based high-throughput sequencing analysis of 53 genes associated with HCTD was performed in all *FBN1*-negative participants. Due to new knowledge, for the follow-up study all the participants were reassessed according to the diagnostic criteria. After reassessment, 84 of the original 105 patients were diagnosed as having MFS according to Ghent-1. The remaining patients were reclassified to other diagnoses. At 10-year follow-up, 16 of 84 were deceased and investigated for causes of death [8]. Of 68 survivors, 47 accepted an invitation to the follow-up study. All participants gave their informed consent prior to inclusion in the study. At follow-up, all the participants received the questionnaire by mail prior to the investigations of the organ manifestations. The questionnaires were completed and returned to the physician who coordinated the investigations at the same day as they were performed. All the participants were investigated for all the features described in the diagnostic criteria with the same methods and modalities as at baseline [6]. The cardiovascular investigations included assessments of mitral valve prolapse; dilatation or dissection of the ascending aorta, the aortic arch and the descending aorta; and dilatation of the main pulmonary artery. For the present analyses new cardiovascular manifestations were defined as: aortic surgery, type A and type B dissection, mitral valve prolapse with and without surgery, endocarditis and stroke. New non-cardiovascular pathology was defined as ectopia lentis, retinal detachment, surgeries due to severe scoliosis, hip surgery and cancer. At follow-up, the participants were interviewed about whether or not they had been followed-up as recommended from the baseline report, and about their use of antihypertensive medication.

Of demographic data, only data on age and sex was obtained.

The Norwegian version 1 of SF-36 was used to assess HRQoL both at baseline and follow-up. Missing data in SF-36 were less than 1% for all the items and there were only single missing items, which were substituted with the subscale mean of the participants according to the SF-36 software procedures.

Previous studies have shown a high validity and reliability of the SF-36 [33, 34]. Minimum clinically important difference (MCID) has been suggested to be between 4 and 6 points for MCS and between 4 and 5 points for PCS [35, 36]. We have chosen a cut-off of 4 points for both MCS and PCS when calculating the proportion of participants that has changed.

### Analysis and statistics

Optum® PRO CoRE software version 1.4.7003.15542 was used to calculate the norm-based scores (mean 50, SD 10) for all eight subscales, MCS and PCS [10]. The norm is based on the 1998 U.S. general population. The norm-based scores were used to calculate within subject changes over time. For comparison of our data with the Norwegian norm population, we calculated z-scores and used a z-score > 0.5 [37] as a measure of clinical significance.

Descriptive statistics are presented as mean values with standard deviation (SD) or proportions. Paired sample *t* tests were performed to compare the means of changes in the eight subscales and MCS and PCS from baseline to 10-year follow-up. The variation in HRQoL changes over 10 years in MFS patients were not known. Hence, a priori sample size calculation was not performed. However, we had estimated that with an SD of eight, we had 90% power to detect the MCID difference of 4 points given a significance level of 5%. In addition, we restricted the multiple regressions to five independent variables.

To explore predictors of changes in the eight subscales and MCS and PCS, we first performed simple linear regression analyses with age, sex, new cardiovascular pathology and non-cardiovascular pathology as predictors, one at a time. Next we performed a total of ten multiple linear regression analyses with the changes in all of the subscales and MCS and PCS as outcome variables, controlling for the baseline score of the outcome variable in addition to age, sex, new cardiovascular pathology and non-cardiovascular pathology. Collinearity diagnostics were used to determine the multicollinearity between the variables.

The results of the regression models are presented with regression coefficients, 95% confidence interval (CI), *R*^2^ and *p* values. *p* ≤ 0.05 was considered statistically significant.

IBM SPSS Statistics for Windows, version 25 (IBM Corp., Armok, NY) was used for the analyses.

## Results

The main characteristics of the MFS cohort are presented in Table 1.

**Table 1:** Characteristics of the MFS cohort at 10-year follow-up, N = 47

Females, n (%)	34 (72.3)
Age, mean (SD)	49.9 (11.7)
*FBN1* mutation (%)	45 (95.7)
Body mass index, mean (SD)	25.3 (5.7)
β-Adrenergic blocking agents or other antihypertensive medication, n (%)	35 (74.5)
Ascending aortic dilatation^a^ at follow-up, n (%)	43 (91.5)
Aortic surgery during life, n (%)	30 (63.8)
New cardiovascular pathology during follow-up, n (%)	21 (44.7)
New non-cardiovascular pathology during follow-up, n (%)	14 (29.8)

^**a**^According to the normal material by Devereux et al. Numbers including patients with aortic graft

In the baseline cohort (N = 84), mean age was 40.2 years (SD 13.2) and the proportion of females was 64%. Mean age of the non-responders at baseline was 34.2 years (SD 13.7), nine males and 12 females. For the baseline cohort, MCS was 46.8 (SD 11.5) and PCS was 40.7 (SD 11.4). For the non-responders, MCS at baseline was 46.5 (SD 11.9) and PCS at baseline was 40.3 (SD 12.8), which is similar to the scores of baseline cohort.

**Fig. 1:**
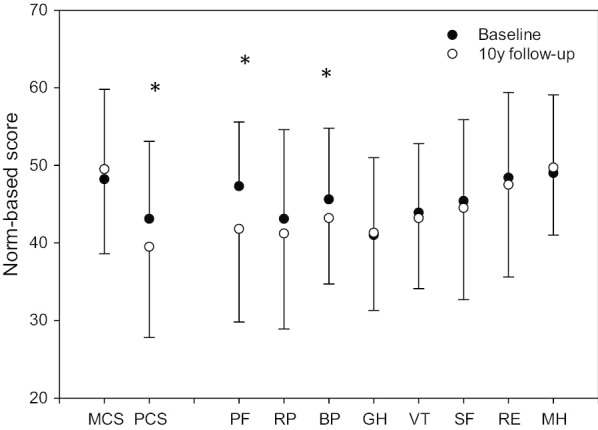
Norm-based scores at baseline and at 10-year follow-up of the Mental Component Summary (MCS), the Physical Component Summary (PCS) and the eight subscales: Physical Functioning (PF), Role-Physical (RP), Bodily Pain (BP), General Health (GH), Vitality (VT), Social Functioning (SF), Role Emotional (RE) and Mental Health (MH).*p < 0.05

Thirty-two percent of the patients did not receive follow-up as recommended from the baseline study. At follow-up statistically significant decline was found in the subscales of physical functioning and bodily pain, with the largest decline in physical functioning, and a statistically significant decline was found for PCS, but not MCS (Fig. 1 and Table 2).

**Table 2:** Raw scores of the eight subscales at baseline, follow-up and changes after 10 years, and norm-based scores of the eight subscales, MCS and PCS at baseline, follow-up and changes after 10 years

	Baseline				Follow-up				Changes after 10 years			
	Raw scores		Norm-based scores		Raw scores		Norm-based scores		Raw scores		Norm-based scores	
	Mean	SD	Mean	SD	Mean	SD	Mean	SD	Mean	SD	Mean	SD
Physical functioning	76.6	19.7	47.3	8.3	63.5	28.6	41.8	12.0	− 5.5	9.5	− 5.5	9.5
Role physical	53.4	40.5	43.1	11.5	46.8	43.5	41.2	12.3	− 1.9	14.8	− 1.9	14.8
Bodily pain	59.9	21.4	45.6	9.2	54.3	19.8	43.2	8.5	− 2.4	7.4	− 2.4	7.4
General health	50.8	21.4	40.9	10.0	51.4	21.3	41.3	10.0	0.3	11.7	0.3	11.7
Vitality	44.0	18.8	43.9	8.9	42.7	19.2	43.2	9.1	− 0.7	9.5	− 0.7	9.5
Social functioning	72.9	24.2	45.4	10.5	71.0	27.2	44.6	11.8	− 0.8	11.4	− 0.8	11.4
Role emotional	78.0	34.9	48.4	11.0	75.2	37.7	47.5	11.9	− 0.9	13.3	− 0.9	13.3
Mental health	73.5	17.7	49.0	10.0	74.7	15.3	49.7	8.7	0.7	9.4	0.7	9.4
MCS			48.2	11.6			49.5	10.9			1.3	10.3
PCS			43.1	10.0			39.5	11.7			− 3.6	10.9

The decline in physical functioning and bodily pain was associated with higher age, *p* < 0.05. The results of the multiple regression analyses showed that none of the factors: sex, new cardiovascular pathology or new non-cardiovascular pathology predicted changes in any of the eight subscales or MCS or PCS at 10-year follow-up. However, older age predicted a larger decline in PCS. For every 1 year increase in age, there is a decrease in the PCS score of 0.33.

**Fig. 2:**
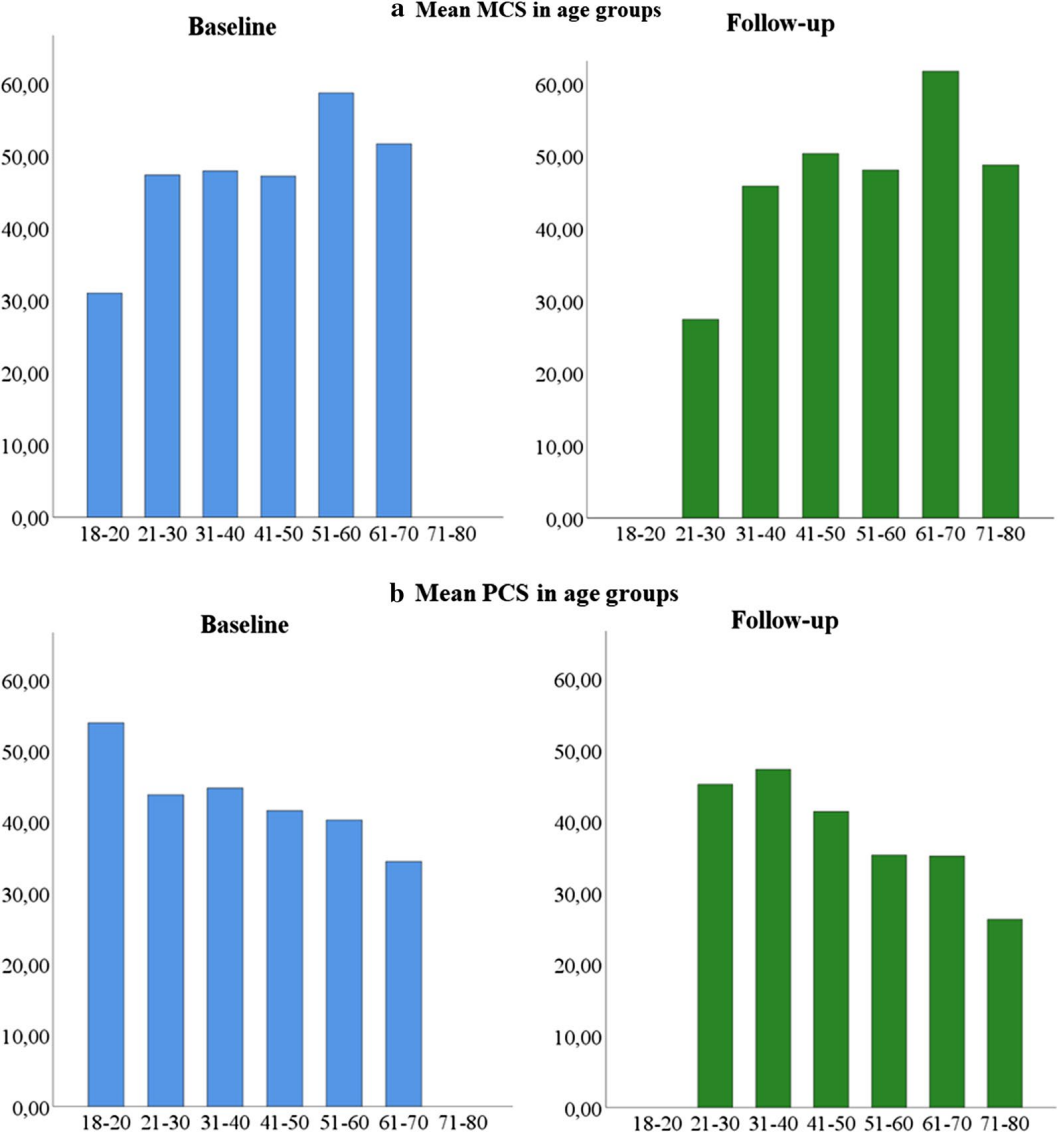
**a** Mean scores of MCS in age groups at baseline and at follow-up, **b** mean scores of PCS in age groups at baseline and at follow-up

None of the MFS related factors predict the change in MCS, still age is the strongest predictor for MCS, showing that for each 1 year increase in age there is an increase in the MCS score of 0.15. However, this finding is not statistically significant. For all subscales as well as MCS and PCS, the decline was related to higher baseline level of the variable (Table 3a, b). This finding is statistically significant for all variables, except for physical functioning, where *p* = 0.054. The mean scores of MCS and PCS at baseline and follow-up in age groups are presented in Fig. 2a, b. When using a MCID of 4 points for both MCS and PCS, nearly half of the study population, 19/47, experienced improved MCS at follow-up, while nearly half, 21/47, experienced reduced PCS at follow-up.

**Table 3:** Multiple regression of the effect of age, sex and new pathology on change in (a) mental component summary (MCS) from baseline to 10-year follow-up, controlling for baseline MCS (R^2^ = 0.30), (b) physical component summary (PCS) from baseline to 10-year follow-up, controlling for baseline PCS (R^2^ = 0.30)

Predictor variable	Regression coefficient, β	95% CI for β		t	*p* value
		Lower bound	Upper bound		
(a)					
Age	0.15	− 0.12	0.41	1.10	0.28
Sex	1.82	− 4.51	8.14	0.58	0.57
New cardiovascular pathology	0.94	− 5.0	6.86	0.32	0.75
New non-cardiovascular pathology	− 0.42	− 6.54	5.70	− 0.14	0.89
MCS	− 0.50	− 0.74	− 0.25	− 4.00	0.000
(b)					
Age	− 0.33	− 0.60	− 0.06	− 2.46	0.02
Sex	− 0.42	− 7.74	6.89	− 0.12	0.91
New cardiovascular pathology	− 3.10	− 9.48	3.27	− 0.98	0.33
New non-cardiovascular pathology	0.47	− 6.04	6.97	0.14	0.89
PCS	− 0.57	− 0.90	− 0.24	− 3.46	0.001

At follow-up, we found significantly reduced scores in all subscales, except mental health, for the MFS cohort compared to the general Norwegian population. Physical functioning z-score − 1.52, role-physical z-score − 0.96, bodily pain z-score − 0.59, general health z-score − 1.01. Vitality z-score − 0.76, social functioning z-score − 0.73 and role-emotional − 0.63.

## Discussion

To our knowledge this is the first long-term study where HRQoL has been reassessed after a 10-year period in a MFS cohort. The follow-up MFS cohort is representative of the baseline cohort regarding age. The proportion of females is higher, and the baseline scores of MCS and PCS is slightly higher than for the baseline cohort. We found decline in the two subscales physical functioning and bodily pain and the PCS scores after 10 years, and that older age at baseline predicted a larger decline in PCS and that older age at baseline is related to decline in physical functioning and bodily pain after 10 years. Physical functioning contributed the most to HRQoL, followed by bodily pain. Only one previous study has presented follow-up data, reporting improved HRQoL on one subscale, role-physical, but the patients were only followed for 1 year [30]. It is unknown whether this improvement in role-physical would sustain after 10 years, and this 1-year follow-up did not show any changes in MCS or PCS.

Raw scores for the eight subscales of SF-36 in the Norwegian general population has been published by Jacobsen et al. [38, 39]. A challenge is the variable level of expected scores dependent on age and gender, rendering comparison between our small MFS sample with the Norwegian reference population difficult. However, a crude comparison between the reference values for the age groups 40–59 years and our population indicate lower scores in MFS patients. In the Norwegian population there is a slight decline in physical functioning and bodily pain from the age group 40–49 years to the age group 50–59 years. In our MFS cohort the decline is from a lower baseline level.

Our MFS cohort has a slightly higher MCS and slightly lower PCS than the ischemic heart disease patients in Huber’s study [40] (MCS 45.9, SD 10.9; PCS 42.3, SD 9.7 in patients < 51 years).

The findings in our study of decline in PCS and no changes in MCS support previous studies which have shown significant lower physical QoL, but no affections of mental QoL [14, 17]. One study even reported slightly better MCS than the general population, but still lower PCS [18]. Only one study has found the opposite result, with lower MCS, but no affections of PCS [15].

The results of this 10-year follow-up indicate that physical limitations might negatively affect HRQoL. Traditionally, MFS patients have had many restrictions regarding physical activity, due to fear of progression of aortic pathology. In recent years, these advice has been moderated. This study supports the use of measures to prevent physical decline and not only focus on organ pathology in the follow-up of MFS patients.

Our findings support other comparable studies which report lower HRQoL in all SF-36 domains compared to healthy controls or the general population [12, 19, 29]. Two studies have only assessed the physical domain of SF-36 and not the mental domain, where one of the studies found reduced physical QoL compared to the general population and the other compared HRQoL between two MFS groups [26, 41].

The results from our study showed that neither new cardiovascular pathology nor new non-cardiovascular pathology predicted changes in HRQoL after 10 years. Only one previous study indicates an association between disease-related factors and HRQoL in MFS patients, but this study has a different design and no follow-up data [27].

No gender differences were found in our study. A study of the general Norwegian population from 2002–2003 reported lower scores for females across almost all eight subscales in all age groups. The minor exceptions were physical functioning for the age group 15–19 years, bodily pain for the age group 20–29 years and general health for those over 79 years, where females had slightly better scores [42]. The same study reported that for both genders, the age groups 40–49 and 60–69 years had the highest scores for role-emotional and mental health, respectively. The younger age groups had the highest PCS scores, which declined with successive age groups. The results of our study show similar findings as for the general Norwegian population regarding decline of physical HRQoL with increasing age. However, the decline is significantly larger in our MFS cohort than for the reference population. Most interestingly, higher age was related to better mental HRQoL at follow-up.

Our study support studies which have reported that the severity of the syndrome does not seem to affect HRQoL [17, 26]. Nearly 45% have developed new cardiovascular manifestations during the 10-year period. These new pathologies did not induce any decline in MCS, which indicate that the severity of the syndrome does not affect the mental health and that MFS patients seem to cope with this aspect of the syndrome well.

One study reported that better HRQoL was associated with insurance and employment status [26]. This is not an important issue for a study of a MFS population in Norway, since national health services are free for all Norwegian residents and a private health insurance is not necessary.

Other papers on QoL in MFS patients have been published, but these are either not HRQoL [43–45] or the study population has been children or young adults with MFS [46, 47]. The results from these studies are not directly comparable to our study, since other methods have been used and none of these studies are long-term follow-up studies.

The strength of our study is that *FBN1* has been sequenced in all patients and all have a confirmed diagnosis of MFS. In addition, all relevant organ systems have been investigated twice, which have given us detailed knowledge about changes of organ manifestations in a 10-year period. Another strength of this study is that all patients completed the questionnaire with less than 1% missing items, and that we do not have any missing data regarding the predictors.

The weakness of this study is the small cohort and the skewness with a higher proportion of females in the baseline cohort. 61% of the drop-outs at follow-up were females. The variation within subject changes were higher than our a priori guess. It is argued by Hoenig and Heisey that post hoc sample size calculations or calculations of detectable effect size do not help in interpretation of the results post hoc [48]. However, the changes in MCS were clearly below the MCID. As MFS is a rare disorder, and the patients were recruited from the Norwegian population of 5.4 million, it was not possible to increase the study cohort. The low sample size was also the reason for restricting the predictors. Lack of analysing the impact of demographic data such as education, profession, socioeconomic status and working status is a clear weakness of the present study. Unfortunately these data were not collected at baseline, since the main focus of the study was to assess organ pathology in MFS. Knowledge of the influence of such factors on HRQoL in a lifetime perspective is needed to provide personalized treatment and follow-up programs. However, due to the low prevalence of MFS multinational studies or registers on much larger cohorts would be needed to obtain such information. One may also discuss whether SF-36 captures the most relevant aspects of life in MFS patients. Living with a chronic and potentially mortal condition may induce a response shift in values compared to the general population. Possibly, education, employment and participation in prioritized life areas may represent more relevant aspects.

## Conclusions

This adult MFS cohort has lower scores in all the domains of the SF-36 compared to the reference population, with a significant decline of HRQoL in the physical domain after 10 years. HRQoL in the mental domain seems to be stable over a 10-year period and gender and development of new organ pathology, including cardiovascular manifestations, does not seem to affect HRQoL. Knowledge of decline in physical HRQoL, not related to organ affections, may be important in the follow-up of MFS patients.

## Data Availability

The dataset used and/or analysed during the current study are available from the corresponding author on reasonable request.

## Abbreviations

*FBN1*: Fibrillin-1 gene
HCTD: Hereditary connective tissue disorders
HRQoL: Health-related quality of life
MCS: The mental component summary
MCID: Minimum clinically important difference
MFS: Marfan syndrome
PCS: The physical component summary
SF-36: Short Form-36 health survey
